# Relative Frequency of *Blastocystis* Subtypes 1, 2, and 3 in Urban and Periurban Human Populations of Arequipa, Peru

**DOI:** 10.3390/tropicalmed5040178

**Published:** 2020-11-27

**Authors:** Kasandra Ascuña-Durand, Renzo S. Salazar-Sánchez, Ricardo Castillo-Neyra, Jorge Ballón-Echegaray

**Affiliations:** 1 Laboratorio de Microbiología Molecular, Facultad de Medicina, Universidad Nacional de San Agustín, 04001 Arequipa, Peru; rendaths@gmail.com; 2 Zoonotic Disease Research Laboratory, One Health Unit, School of Public Health and Administration, Universidad Peruana Cayetano Heredia, 4314 Lima, Peru; cricardo@upenn.edu; 3 Department of Biostatistics, Epidemiology & Informatics, Perelman School of Medicine of the University of Pennsylvania, Pennsylvania, PA 19104, USA; 4 Departamento de Microbiología y Patología, Facultad de Medicina, Universidad Nacional de San Agustín, Santa Catalina 117, 04001 Arequipa, Peru

**Keywords:** *Blastocystis*, protozoon, controversial, epidemiological variables, symptoms

## Abstract

*Blastocystis* is one of the most common protozoa found in the human gut and are genetically diverse and widely distributed around the world. Nonspecific and inconsistent symptoms have been associated with this protozoon; thus, its clinical importance remains controversial. Our aim was to estimate the relative frequency of *Blastocystis* subtypes 1, 2, and 3, which are the predominant subtypes reported in South America, based on conserved regions of SSU rDNA sequences and determine the factors associated with them. A total of 116 *Blastocystis*-positive stool samples were processed using conventional PCR with *Blastocystis*-specific primers. We identified subtype 1 (10.3%), subtype 2 (7.8%), subtype 3 (25.0%), and mixed subtype infections (8.7%). However, we could not identify any *Blastocystis* subtypes in 48.3% of the samples; therefore, it is likely that other subtypes were present in the area. No association was found between any gastrointestinal symptom and single or mixed *Blastocystis* subtypes. We found a statistically significant association between *Blastocystis* subtype 2 and irritable bowel syndrome (OR = 17.8, 95% CI = 1.5–408.4, *p* = 0.039); however, the number of samples with IBS was small (*n* *=* 4). There was no association between the *Blastocystis* subtypes and any epidemiological variable studied. In rural populations, we only identified subtype 1, while in urban and periurban populations, we identified subtypes 1, 2, and 3.

## 1. Introduction

*Blastocystis* is a protozoon found in the gut of humans and animals [1]. This microorganism is genetically diverse, with 17 known subtypes [1] distributed throughout the world [2]. Subtypes 1–9 are found in both humans and animals, subtype 9 is only found in humans [3], and subtypes 10–17 are found in domestic and wild animals [4]. Subtypes 1, 2, and 3 are the most common subtypes that are associated with gastrointestinal symptoms [5,6] and the most prevalent in South America [7]; subtype 4 is mainly found in Europe and Asia [6]. 

Over the last decade, *Blastocystis* has been studied widely, but it remains unclear whether it is pathogenic [8]. Some studies report an association between *Blastocystis* and gastrointestinal symptoms, such as abdominal pain, diarrhea, vomiting, constipation, and irritable bowel syndrome (IBS) [5,9,10], whereas other investigations failed to prove any correlation between *Blastocystis* and health issues [11,12] or suggest that *Blastocystis* could be considered normal or beneficial for the microbiota [13,14]. *Blastocystis* transmission is generally associated with poor access to healthcare and unsanitary living conditions around the world [3,15].

In South America, there have been some studies focused on this protozoon [2,16,17,18], with only one study of *Blastocystis* subtypes in Peru [7]. However, *Blastocystis* infection has been reported in some Peruvian cities [19,20,21] and Arequipa has had the highest prevalence levels with 48.3% in elementary school children and 81.9% in low-income communities [22,23]. Our primary aim was to estimate the relative presence of South America’s most common subtypes of *Blastocystis* (subtypes 1, 2, and 3) in urban and periurban human populations in Arequipa city, Peru. We also assessed the association of these subtypes with gastrointestinal symptoms and sanitary living conditions.

## 2. Materials and Methods 

### 2.1. Ethical Statement 

The protocol for this study was reviewed and approved by the Institutional Review Boards of the Universidad Peruana Cayetano Heredia (reference number 18006). Written informed consent was provided by all participants prior to the study. Written assent and parents’ informed consent was provided for minors under the age of 18.

### 2.2. Study Site

Arequipa city is the second-most populous city in Peru, with approximately a million inhabitants, located in the country’s southern highlands at 2400 meters above sea level [24]. The sprawling growth of the city in the last few decades established two kinds of well-defined areas: periurban and urban. Periurban areas grew around the city with limited access to basic services, such as water, sanitation, health, and education, whereas urban areas have comprehensive access to these services. Thus, the socioeconomic status of participants from periurban areas is generally lower than that of urban participants [24,25]. Arequipa is also surrounded by a widely rural area with similar sanitary features to the periurban areas. 

### 2.3. Study Population

We obtained stool samples from 116 *Blastocystis*-infected participants from different districts of Arequipa, both with and without gastrointestinal symptoms, both adults and children, and from both sexes. The recruitment method was described elsewhere [26]. All participants completed epidemiological surveys to assess their clinical symptoms and sanitary living conditions.

### 2.4. Detection of Blastocystis

Each participant’s sample was collected in a sterilized plastic mouth flask without any additive. All participants were instructed about the correct procedure to collect their stool sample. Those instructions were given in a short letter to each participant. At that point, we answered any questions that the participants had. 

All stool samples were analyzed using a rapid concentration saline solution. We examined the pellet under light microscopy at 400× magnification and confirmed with blue methylene-stained stool smear under 1000× magnification, as was explained previously [26]. *Blastocystis*-positive samples were aliquoted in cryovials and stored at −80 °C.

### 2.5. DNA Extraction and PCR Amplification

We used 200 mg of stool samples stored at −80°C to extract DNA using the Norgen Stool DNA Isolation Kit (Norgen, Biotek Corporation, Thorold, ON, Canada). PCR was performed with 25 μL of 2X PCR Taq Plus MasterMix (Applied Biological Materials Inc. ABM, Richmond, BC, Canada), 100 ng (~2 ng/μL) of a DNA template, and 200 nM of *Blastocystis*-specific primers based on the conserved regions of SSU rDNA sequences: F:5′-GAAGGACTCTCTGACGATGA-3’/R:5′-GTCCAAATGAAAGGCAGC-3’ (351 bp) for subtype 1 [5], F:5′-CATGAGTAAAGTCCCGTWGGGA-3’/R:5′-CCCTTTTACAGTTCATTCGCCTA-3’ (1500 bp) for subtype 2 [27], and F:5′-TAGGATTTGGTGTTTGGAGA-3’/R:5′-TTAGAAGTGAAGGAGATGGAAG-3’ (526 bp) for subtype 3 [5], obtaining a final volume of 50 μL. The PCR parameters were standardized experimentally with reference to the setting parameters used by the authors of the primers [5,27] and adjusted according to the amplification kit manufacturer’s specifications for use under local conditions and equipment. The PCR conditions used for *Blastocystis* subtypes 1 and 3 were one cycle of initial denaturation at 94 °C for 3 min, followed by 35 cycles for denaturation at 94 °C for 30 s, annealing at 54 °C for subtype 1 and 57 °C for subtype 3, extension at 72 °C for 60 s, and an additional cycle for the final extension at 72 °C for 5 min. For subtype 2, the PCR conditions were one cycle of initial denaturation at 94 °C for 5 min, followed by 35 cycles for denaturation at 94 °C for 30 s, annealing at 58 °C for 50 s, extension at 73 °C for 90 s, and an additional cycle for the final extension at 73 °C for 7 min. Electrophoresis was performed by adding 8 µL of the PCR products to a 1.7% agarose gel and staining with GelRed^®^ Nucleic Acid Gel Stain, 10,000× in Water (Biotium Inc., Fremont, CA, USA) for 30 min at 100 V. *Blastocystis* samples were considered positive for a subtype when a clear visible band of the correct size for the primer pairs were observed in the gel under UV light.

### 2.6. Statistical Analysis

Descriptive analysis was used to explore *Blastocystis* subtypes frequencies per study area, the composition of subtypes within households, and other variables of interest, such as household sanitary conditions, demographics, and food consumption behaviors. The association of *Blastocystis* subtypes with gastrointestinal symptoms and sanitary living conditions were analyzed using chi-square and Fisher’s exact tests. The association between *Blastocystis* subtypes and the patients’ ages was analyzed using the Kruskal–Wallis test. For the epidemiological analysis, we selected variables that could explain the possible source of *Blastocystis* subtypes, as were suggested in previous studies [28,29]. Odds ratios (ORs) and 95% confidence intervals were used to estimate the association between specific *Blastocystis* subtypes and potential risk factors. We also explored the spatial distribution of subtypes in the city. All analyses were conducted using R 3.6.2 [30].

## 3. Results

We obtained DNA from 116 *Blastocystis-*positive individuals, 50 of which were co-infected with other protozoa. The participants’ ages ranged from 2 to 82 years old and 50.4% were female. Out of the 116 stool samples analyzed, 61.2% came from periurban areas, 33.6% came from urban areas, and 5.2% from rural areas.

**Figure 1:**
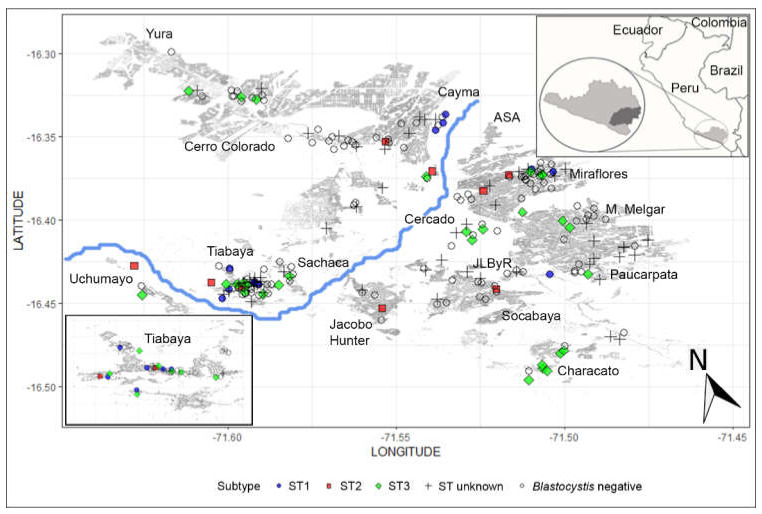
Spatial distribution of the *Blastocystis* subtypes. All urban, periurban, and rural districts in Arequipa city are shown.

Participants were enrolled from urban, periurban, and rural areas across the city (Figure 1). The spatial distribution of subtypes shows that the presence of *Blastocystis* subtype 3 was the most prevalent subtype, was found across the city, and was the only one subtype identified in many districts. *Blastocystis* subtype 2 was the only subtype identified in two districts. *Blastocystis* subtypes other than 1, 2, or 3 (unknown subtype) were present in all areas across the city. Tiabaya district, located in the southwestern area of the city where sewage water is disposed into the river, showed the presence of *Blastocystis* subtypes 1, 2, and 3, including unknown subtypes. The map also shows a concentration of unknown subtypes in the northeastern part of the city. 

**Figure 2:**
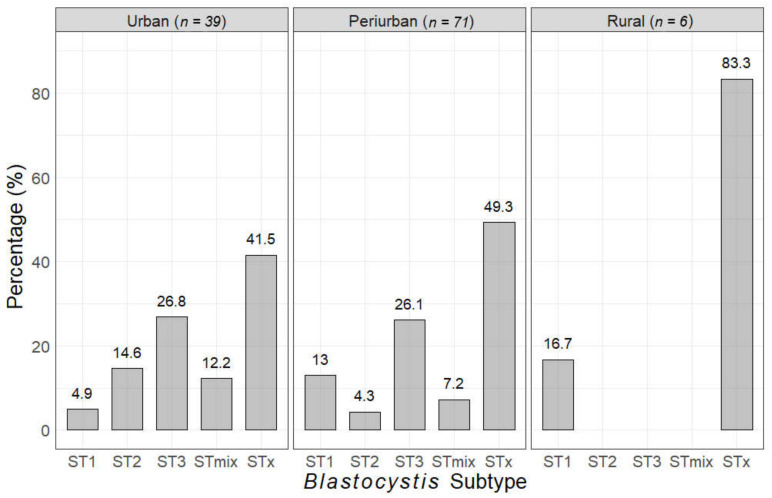
*Blastocystis* subtype distribution in each study area. STx corresponds to an unknown *Blastocystis* subtype and STmix corresponds to mixed subtypes.

We identified 43.1% of samples with single subtype infections and 8.7% samples with mixed subtype infections. *Blastocystis* subtype 3 was the most prevalent in the study sample (25.0%), followed by subtypes 1 (10.3%) and subtype 2 (7.8%). The most common mixed subtypes identified were subtypes 1 and 3 (7.8%), followed by subtypes 1, 2, and 3 (0.9%) (Table 1). We could not identify *Blastocystis* subtypes 1, 2, or 3 in almost half of the samples (48.3%). The median age with single-subtype infections was 36.5 years old, whereas the median age with mixed infections was 16.5. Figure 2 shows the frequency of *Blastocystis* subtypes in each study area, with *Blastocystis* subtype 3 being the most frequent in both urban and periurban areas (28.2% and 25.4%, respectively). Subtype 2 was the least frequent in periurban areas (4.2%) and subtype 1 was the least frequent in urban areas (2.6%); only subtype 1 was identified in participants from rural localities (Figure 2).

**Figure 3:**
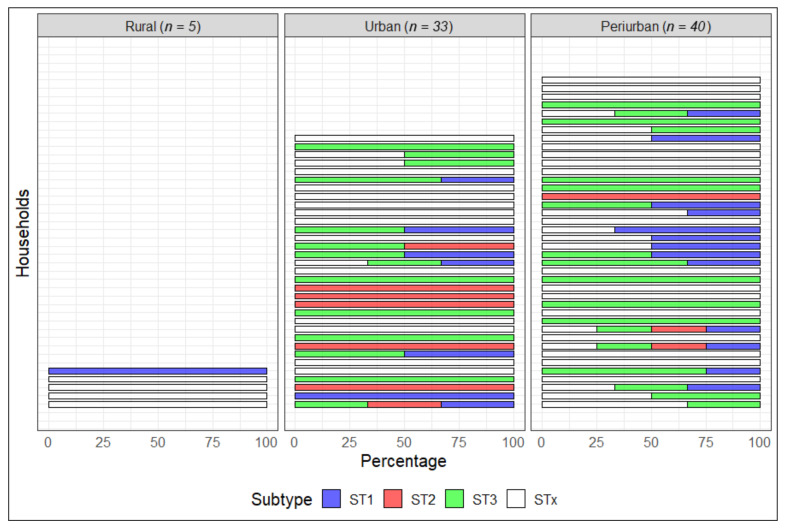
Distribution and composition of *Blastocystis* subtypes per household and study area. * STx: unknown *Blastocystis* subtype. *n* corresponds to the number of households that participated in each study area and not the number of participants. The samples came from 116 people from 78 households within 3 areas.

Figure 3 shows the composition of *Blastocystis* subtypes 1, 2, and 3, as well as unknown *Blastocystis* subtypes in each household (*n* = 78 households), where the most common composition involved subtypes 1 and 3 with 10.3% (8/78 households). Twenty households presented with only one subtype (ST1 *=* 2, ST2 = 6, ST3 = 12), where subtype 3 was most prevalent in urban and periurban areas (18/42 and 14/33 respectively). A total of 61.5% of households had an unknown subtype: urban 15/33, periurban 29/42, and rural 4/5. Additionally, we observed the presence of the three *Blastocystis* subtypes (1, 2, and 3) in one participant from an urban household, and the presence of the three subtypes and unknown subtype(s) in one periurban household but in four different household members. We also identified other intestinal protozoa in the stool samples. The different coinfection combinations between *Blastocystis* subtypes and other protozoa are detailed in Table S1.

**Figure 4:**
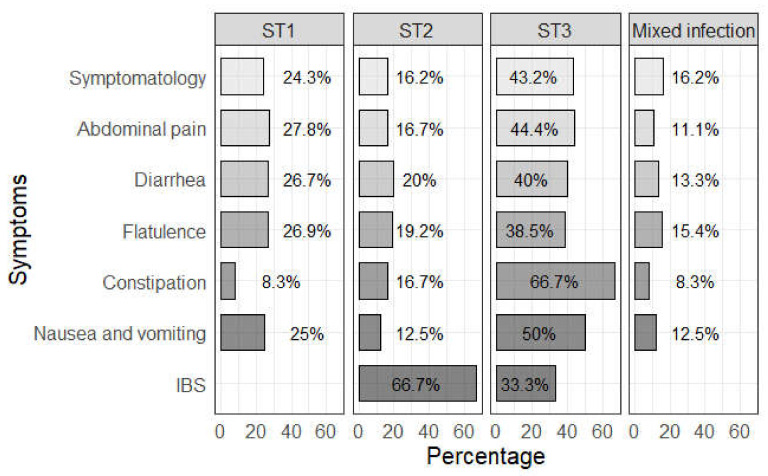
Symptomatology percentage by *Blastocystis* subtypes 1, 2, 3, and mixed infection.

In order to exclude intestinal symptoms and disorders caused by other protozoa, the statistical analysis of clinical manifestations was performed only with *Blastocystis*-positive participants who were negative to other microorganisms. Fisher’s exact test showed no association between single-subtype infections and individual gastrointestinal symptoms. However, there was a statistically significant association between *Blastocystis* subtype 2 and IBS (OR = 17.8, 95% CI = 1.5–408.4, *p* = 0.039). Figure 4 shows the frequency of gastrointestinal symptoms by single- and mixed-subtype infections.

In the analysis, we considered the mixed subtype infections (*n* = 10) as an additional subtype group to compare, though we excluded the rural area from the analysis because we had only one participant in that category. We did not find any statistical association between the participants’ age and infection with specific *Blastocystis* subtypes (*p* = 0.993). We also did not find any statistically significant association between *Blastocystis* subtypes, either single or mixed, and sanitary living conditions (Table 2). 

## 4. Discussion

This study determined the presence and frequency of *Blastocystis* subtypes 1, 2, and 3 in Arequipa, Peru, at a molecular level, analyzing their relationships with clinical and epidemiological findings in urban and periurban areas in the city. The presence of subtype 3 and an unknown subtype in Peru was previously reported in a molecular study based on 13 participants [7]. In our study, we used a much larger sample size, but we only looked for subtypes 1, 2, and 3. We found that *Blastocystis* subtype 3 was the most prevalent, followed by subtypes 1 and 2. Similar prevalence were reported in other study sites in South America [2,7,31] and other places around the world [5,32,33]. However, other studies from Brazil and Lao People’s Democratic Republic reported that subtype 1 was the most prevalent in their study areas [34,35], whilst in Europe, the most prevalent subtypes were 3 and 4 [36,37].

Regarding the high number of non-identified *Blastocystis* subtypes in half of the samples, it is possible that other *Blastocystis* subtypes that are less reported in surrounding countries (different than 1, 2, and 3) were more prevalent in this area of Peru [7]. Furthermore, the high prevalence of more than three *Blastocystis* subtypes circulating in human Peruvian populations increased the chances of different combinations of mixed subtype infection between subtypes 1, 2, 3, and others. These mixed infections could lead to low accuracy in the amplification process for primer competition or inhibition for the presence of the whole stool DNA in the sample [5,27], which made the subtype identification difficult to achieve. Other possible explanations are related to a novel *Blastocystis* subtype; however, these hypotheses should be studied in detail.

Varying prevalence of dual-mixed subtype infections between *Blastocystis* subtypes 1, 2, and 3 were reported in Brazil, Turkey, and Iran [5,32,34]. Here, we report dual-mixed subtype infections between subtypes 1 and 3. We also report a triple-mixed subtype infection (1, 2, and 3) in only one sample. An influx of other subtypes into Arequipa could be caused by job migration [38,39,40,41], tourism [33], or even zoonotic transmission due to farming activities [2]. For this reason, it is necessary to carry out more studies to understand the relative frequency of other subtypes that are less common in other countries.

We assumed that human migration in Arequipa city established a complex mixing of subtypes between periurban and urban areas across the city. Unfortunately, we could not extend this finding to rural areas because we had few participants from this area. Our findings also showed clustering in the distribution of subtypes between rural and urban/periurban areas. However, the wide spatial distribution of subtypes all over Arequipa city contrasted with the distribution of subtypes in Turkey, where subtypes 1, 2, and 3 were restricted to cities and subtypes 5, 6, and 7 occurred in less urban areas [42]. Other factors or conditions that allow or facilitate the transmission of *Blastocystis* subtypes across communities and districts remain unknown.

There is limited available research about the role of sanitary conditions on transmission routes and *Blastocystis* sources. Only subtype 3 has been associated with drinking unpurified water [29]. Other variables, such as food consumption and raising domestic animals, have not been reported to be associated with subtypes 1 and 3 [43]. We did not find any association between sanitary conditions nor the presence of animals nor fresh vegetables and fruit consumption and subtypes 1, 2, or 3 of *Blastocystis*. These findings were similar to those from Spain, which showed no association with drinking water or hand- or food-washing behavior [44]. However, in rural areas, socioeconomic status and food or water contamination are associated with exposure to livestock and wildlife, which could explain the reported distribution of specific subtypes in rural communities of Turkey [42]. General prevalence studies on intestinal parasite infections have been associated with the presence of *Blastocystis* infection and untreated water consumption, feces disposal practices, and inadequate hand-washing habits [21,45]. There is little literature available related to how dietary practices influence the presence of *Blastocystis* in the human gut. However, this factor should be studied because it has previously been found to be an important factor that affects *Blastocystis* prevalence, subtypes, and the pathogenicity linked to gut microbiota [46,47]. Our results showed no association.

Demographic characteristics were explored as another factor that could be linked to the distribution of *Blastocystis* subtypes. A study from Rio de Janeiro, Brazil, reported statistical differences between the prevalence of *Blastocystis* in men and women, and proposed that economic activities could explain this finding [2]. Contrary to these results, we did not find any statistical association between *Blastocystis* subtypes and sex, despite many adult women working in the household in our study population, which could increase the risk of infection due to food contamination [45,48], lack of sanitation [2], and domestic animal handling in periurban communities of Arequipa [39]. On the other hand, age is another important demographic factor associated with infection and plays a key role in intestinal infections, as described in other studies [5,42,49]. We found that participants with mixed infections were younger overall than participants with single infections. These results suggest that a person can get infected with different subtypes at earlier ages, but over time, one subtype might outcompete the others, creating a pattern associated with age. More studies must be performed to elucidate this hypothesis.

*Blastocystis* pathogenicity was suggested in 1986 [50]. Nowadays, some studies suggest that subtypes 1 and 3 are responsible for discrete gastrointestinal symptoms, such as abdominal pain, diarrhea, vomiting, and flatulence [5,9], which we did not find in our study. However, our data suggested a statistical association between subtype 2 and IBS, but these results should be interpreted with caution since the sample size for that analysis was low (only four participants showing IBS). These findings are similar to previous reports in Turkey, Iran, and Mexico, where subtypes 1, 2, and 3 were found in patients with IBS [51,52,53]. Additionally, another study in Chile, reported that *Blastocystis* subtypes 1, 2, and 4 are related to IBS [16].

An important limitation of our study was not considering the potential presence of subtypes reported at lower proportions in South America and expecting similar subtype frequencies as those previously reported in Peru [7]. Future studies could benefit from applying other molecular techniques, such as sequencing, to elucidate the relative frequency of other *Blastocystis* subtypes that are less common in this population. We also recommend exploring the likely association between subtypes 2 and 3 with IBS in murine models to discard this pseudo-association or conducting a case-control study with IBS patients. Our study contributes to a better understanding of the molecular epidemiology of this protozoon and its potential impact on clinical manifestations. Our findings also form the basis for future research and developing new hypotheses about the distribution and clinical manifestations of *Blastocystis* subtypes in Peru and Latin America.

## 5. Conclusions

We report the presence of *Blastocystis* subtypes 1, 2, and 3 in rural, urban, and periurban areas of Arequipa city in one of the few molecular studies of this protozoon conducted in South America. Even though subtypes 1, 2, and 3 are the most common worldwide and in South America, in our study population in Arequipa, they only accounted for 51.8% of the *Blastocystis*-positive samples, suggesting the presence of other highly prevalent subtypes that should be studied. No significant association was found between sanitary living conditions and any specific *Blastocystis* subtypes. 

## Figures and Tables

**Table 1:** Frequency and prevalence of *Blastocystis* subtypes 1, 2, and 3 (ST1, ST2, and ST3) and the type of infection identified in the tested samples.

Type of *Blastocystis* Infection	*Blastocystis* Subtype (*n*)	Prevalence (%)	95% CI
Simple infection	ST1 (12)	10.3	4.3–16.3
	ST2 (9)	7.8	2.8–12.8
	ST3 (29)	25	17.0–33.0
	Unknown (56)	48.3	39.3–57.3
Mixed infection	ST1,3 (9)	7.8	2.8–12.8
	ST1,2,3 (1)	0.8	0–2.9

**Table 2:** Relationship between *Blastocystis* subtypes and sanitary living conditions.

	*Blastocystis* Subtype				
Variable	ST1	ST2	ST3	Mixed Infection	*p*-Value
	*n =* 21	*n =* 10	*n =* 39	*n =* 10	
Female	16 (76%)	5 (50%)	21 (54%)	7 (70.0%)	0.304 ^†^
Age					
Mean (SD)	32.5 (23%)	40.1 (23%)	37.3 (23%)	31 (26%)	0.995 ^&^
Median (IQR)	33 (11–49%)	46 (18–57%)	33 (14–54%)	16 (10–50%)	
Areas					
Urban	6 (29%)	7 (70%)	16 (41%)	5 (50%)	0.178 ^†^
Periurban	15 (71%)	3 (30%)	23 (59%)	5 (50%)	
Water supply					
Tap water	20 (95%)	10 (100%)	36 (92%)	10 (100%)	1 ^†^
Not tap water	1 (5%)		3 (8%)		
Final feces disposal					
Piped sewer system	20 (95%)	10 (100%)	35 (90%)	10 (100%)	0.761 ^†^
Latrine	1 (6%)		4 (10%)		
Presence of animals	15 (71%)	9 (90%)	32 (82%)	7 (70%)	0.554 ^†^
Presence of dogs	12 (57%)	8 (80%)	26 (67%)	6 (60%)	0.631 ^†^
Presence of cats	5 (24%)	2 (20%)	12 (31%)	2 (20%)	0.874 ^†^
Presence of guinea pigs	3 (14%)	1 (10%)	10 (26%)	2 (20%)	0.725 ^†^
Presence of rabbits	5 (24%)	2 (20%)	9 (23%)	2 (20%)	1 ^†^
Presence of poultry	5 (24%)	2 (20%)	14 (36%)	2 (20%)	0.672 ^†^
Presence of flies	15 (71%)	6 (60%)	29 (74%)	7 (70%)	0.831 ^†^
Presence of cockroaches	5 (24%)	1 (10%)	9 (23%)	2 (20%)	0.911 ^†^
Presence of rodents	3 (14%)	2 (20%)	7 (18%)	1 (10%)	1 ^†^
Place of food consumption					
House	17 (81%)	7 (70%)	32 (82%)	8 (80%)	0.377 ^†^
Restaurant	2 (10%)	1 (10%)	1 (3%)	1 (10%)	
House and restaurant		2 (20%)	4 (10%)		
House and food cart	2 (10%)		2 (5%)	1 (10%)	
Kind of water consumption					
Boiled water	18 (86%)	8 (80%)	32 (82%)	9 (90%)	0.737 ^†^
Tap water	1 (5%)				
Both	2 (10%)	2 (20%)	7 (18%)	1 (10%)	
Fresh vegetables consumption	8 (38%)	5 (50%)	14 (36%)	4 (40%)	0.904 ^†^
Fresh fruits consumption	10 (48%)	5 (50%)	16 (41%)	6 (60%)	0.755 ^†^

^†^ Fisher’s exact test. ^&^ Kruskal–Wallis test.

## Supplementary Materials

The following are available online at https://www.mdpi.com/2414-6366/5/4/178/s1, Table S1. Blastocystis Subtypes Coinfection with Other Intestinal Protozoa.

## References

[1] Stensvold C.R., Suresh G.K., Tan K.S., Thompson R.A., Traub R.J., Viscogliosi E., Yoshikawa H., Clark C.G. (2007). Terminology for Blastocystis subtypes—A consensus. Trends Parasitol..

[2] Barbosa C.V., Barreto M.M., Andrade R.D.J., Sodré F., D’Avila-Levy C.M., Peralta J.M., Igreja R.P., De Macedo H.W., Santos H.L.C. (2018). Intestinal parasite infections in a rural community of Rio de Janeiro (Brazil): Prevalence and genetic diversity of Blastocystis subtypes. PLoS ONE.

[3] Yoshikawa H., Tokoro M., Nagamoto T., Arayama S., Asih P.B., Rozi I.E., Syafruddin D. (2016). Molecular survey of Blastocystis sp. from humans and associated animals in an Indonesian community with poor hygiene. Parasitol. Int..

[4] Stensvold C.R., Clark C.G. (2016). Current status of Blastocystis: A personal view. Parasitol. Int..

[5] Khademvatan S., Masjedizadeh R., Yousefi-Razin E., Mahbodfar H., Rahim F., Yousefi E., Foroutan M. (2018). PCR-based molecular characterization of Blastocystis hominis subtypes in southwest of Iran. J. Infect. Public Health.

[6] Alfellani M.A., Stensvold C.R., Vidal-Lapiedra A., Onuoha E.S.U., Fagbenro-Beyioku A.F., Clark C.G. (2013). Variable geographic distribution of Blastocystis subtypes and its potential implications. Acta Trop..

[7] Ramírez J.D., Sánchez A., Hernández C., Flórez C., Bernal M.C., Giraldo J.C., Reyes P., López M.C., García L., Cooper P.J. (2016). Geographic distribution of human Blastocystis subtypes in South America. Infect. Genet. Evol..

[8] Skotarczak B. (2018). Genetic diversity and pathogenicity of Blastocystis. Ann. Agric. Environ. Med..

[9] Kaneda Y., Horiki N., Cheng X.J., Fujita Y., Maruyama M., Tachibana H. (2001). Ribodemes of Blastocystis hominis isolated in Japan. Am. J. Trop. Med. Hyg..

[10] Ramírez J.D., Sánchez L.V., Bautista D.C., Corredor A.F., Flórez A.C., Stensvold C.R. (2014). Blastocystis subtypes detected in humans and animals from Colombia. Infect. Genet. Evol..

[11] Cifre S., Gozalbo M., Ortiz V., Soriano J.M., Merino-Torres J.F., Trelis M. (2018). Blastocystis subtypes and their association with Irritable Bowel Syndrome. Med. Hypotheses.

[12] Di Cristanziano V., D’alfonso R., Berrilli F., Sarfo F.S., Santoro M., Fabeni L., Knops E., Heger E., Kaiser R., Dompreh A. (2019). Lower prevalence of Blastocystis sp. infections in HIV positive compared to HIV negative adults in Ghana. PLoS ONE.

[13] Andersen L.O., Bonde I., Nielsen H.B., Stensvold C.R. (2015). A retrospective metagenomics approach to studyingBlastocystis. FEMS Microbiol. Ecol..

[14] Andersen L.O., Karim A.B., Roager H.M., Vigsnæs L.K., Krogfelt K.A., Licht T.R., Stensvold C.R. (2016). Associations between common intestinal parasites and bacteria in humans as revealed by qPCR. Eur. J. Clin. Microbiol. Infect. Dis..

[15] Del Coco V.F., Molina N.B., Basualdo J.A., Córdoba M.A. (2017). *Blastocystis* spp.: Avances, controversias y desafíos futuros. Rev. Argent. Microbiol..

[16] Peña S., Carrasco G., Rojas P., Castillo D., Ozaki L.S., Mercado R. (2020). Determination of subtypes of Blastocystis sp. in Chilean patients with and without inflammatory bowel syndrome, A preliminary report. Parasite Epidemiol. Control..

[17] Jiménez P.A., Jaimes J.E., Ramírez J.D. (2019). A summary of Blastocystis subtypes in North and South America. Parasites Vectors.

[18] Ramírez J.D., Flórez C., Olivera M., Bernal M.C., Giraldo J.C. (2017). Blastocystis subtyping and its association with intestinal parasites in children from different geographical regions of Colombia. PLoS ONE.

[19] Ibáñez-Herrera N., Jara C.C., Guerra M.A., Díaz-Limay E. (2004). Prevalencia del enteroparasitismo en escolares de comunidades nativas del Alto Marañón, Amazonas, Perú. Rev. Peru Med. Exp. Salud Publica.

[20] Sánchez L., Gallardo J., Jara C. (2011). Prevalencia de Infección por Blastocystis y Protozoarios Intestinales en Niños de “Alto Trujillo”, La Libertad, Perú. Sciéndo.

[21] Choi B., Kim B. (2017). Prevalence and Risk Factors of Intestinal Parasite Infection among Schoolchildren in the Peripheral Highland Regions of Huanuco, Peru. Osong Public Health Res. Perspect..

[22] Quispe-Juli C.U., Coila Y.S.C., Moreno-Loaiza O. (2016). Elevada prevalencia de *Blastocystis* spp. en niños de una escuela periurbana. An. Fac. Med..

[23] Casquina-Guere L., Martínez-Barrios E. (2011). Prevalencia y epidemiología del parasitismo intestinal en escolares de nivel primario de Pucchún, Camaná, Arequipa, Perú. Neotrop. Helminthol..

[24] Instituto Nacional de Estadística e Informática—INEI (2018). Censos Nacionales 2017: XII de Población, VII de Vivienda y III de Comunidades Indígenas. Lima—Perú. https://www.inei.gob.pe/media/MenuRecursivo/publicaciones_digitales/Est/Lib1551/.

[25] Leclerc A., Kaminski M., Lang T. (2009). Combler le fossé en une génération: Le rapport de l’OMS sur les déterminants sociaux de la santé. Rev. D’Épidémiol. Santé Publique.

[26] Sanchez R.S.S., Ascuña-Durand K., Castillo-Neyra R., Vásquez-Huerta V., Martínez-Barrios E., Ballón-Echegaray J. (2020). Socio-demographic determinants associated with Blastocystis infection in Arequipa, Peru. MedRxiv.

[27] Yoshikawa H., Iwamasa A. (2016). Human Blastocystis subtyping with subtype-specific primers developed from unique sequences of the SSU rRNA gene. Parasitol. Int..

[28] Minvielle M.C., Pezzani B.C., Cordoba M.A., De Luca M.M., Apezteguia M.C., Basualdo J.A. (2004). Epidemiological survey of *Giardia* spp. and Blastocystis hominis in an Argentinian rural community. Korean J. Parasitol..

[29] Noradilah S.A., Moktar N., Anuar T.S., Lee I.L., Salleh F.M., Manap S.N.A.A., Mohtar N.S.H.M., Azrul S.M., Abdullah W.O., Nordin A. (2017). Molecular epidemiology of blastocystosis in Malaysia: Does seasonal variation play an important role in determining the distribution and risk factors of Blastocystis subtype infections in the Aboriginal community?. Parasites Vectors.

[30] R Project. What Is R?. http://www.r-project.org/about.html.

[31] Villamizar X., Higuera A., Herrera G., Vasquez-A L.R., Buitron L., Muñoz L.M., Gonzalez-C F.E., Lopez M.C., Giraldo J.C., Ramírez J.D. (2019). Molecular and descriptive epidemiology of intestinal protozoan parasites of children and their pets in Cauca, Colombia: A cross-sectional study. BMC Infect. Dis..

[32] Dogan N., Aydin M., Tuzemen N.U., Dinleyici E.C., Oguz I., Dogruman-Al F. (2017). Subtype distribution of *Blastocystis* spp. isolated from children in Eskisehir, Turkey. Parasitol. Int..

[33] Rojas-Velázquez L., Moran P., Serrano-Vázquez A., Fernández L.D., Pérez-Juárez H., Poot-Hernandez A.C., Portillo-Bobadilla T., Gonzalez E., Hernandez E., Partida-Rodriguez O. (2018). Genetic Diversity and Distribution ofBlastocystisSubtype 3 in Human Populations, with Special Reference to a Rural Population in Central Mexico. BioMed Res. Int..

[34] Malheiros A.F., Stensvold C.R., Braga G.B., Shaw J.J., Clark C.G. (2011). Molecular Characterization of Blastocystis Obtained from Members of the Indigenous Tapirapé Ethnic Group from the Brazilian Amazon Region, Brazil. Am. J. Trop. Med. Hyg..

[35] Sanpool O., Laymanivong S., Thanchomnang T., Rodpai R., Sadaow L., Phosuk I., Maleewong W., Intapan P.M. (2017). Subtype identification of human *Blastocystis* spp. isolated from Lao People’s Democratic Republic. Acta Trop..

[36] Stensvold C.R., Christiansen D.B., Olsen K.E.P., Nielsen H.V. (2011). Blastocystis sp. Subtype 4 is Common in Danish Blastocystis-Positive Patients Presenting with Acute Diarrhea. Am. J. Trop. Med. Hyg..

[37] Forsell J., Granlund M., Stensvold C.R., Clark G.C., Evengård B. (2012). Subtype analysis of Blastocystis isolates in Swedish patients. Eur. J. Clin. Microbiol. Infect. Dis..

[38] Castillo-Neyra R., Toledo A.M., Arevalo-Nieto C., Macdonald H., De la Puente-León M., Naquira-Velarde C., Paz-Soldan V.A., Buttenheim A.M., Levy M.Z. (2019). Socio-spatial heterogeneity in participation in mass dog rabies vaccination campaigns, Arequipa, Peru. PLoS Negl. Trop. Dis..

[39] Levy M.Z., Bowman N.M., Kawai V., Waller L.A., Del Carpio J.G.C., Benzaquen E.C., Gilman R.H., Bern C. (2006). Periurban Trypanosoma cruzi–infected Triatoma infestans, Arequipa, Peru. Emerg. Infect. Dis..

[40] Bowman N.M., Kawai V., Levy M.Z., Del Carpio J.G.C., Cabrera L., Delgado F., Malaga F., Benzaquen E.C., Pinedo V.V., Steurer F. (2008). Chagas Disease Transmission in Periurban Communities of Arequipa, Peru. Clin. Infect. Dis..

[41] Bayer A.M., Hunter G.C., Gilman R.H., Del Carpio J.G.C., Naquira C., Bern C., Levy M.Z. (2009). Chagas Disease, Migration and Community Settlement Patterns in Arequipa, Peru. PLoS Negl. Trop. Dis..

[42] Koltas I.S., Eroglu F. (2016). Subtype analysis of Blastocystis isolates using SSU rRNA-DNA sequencing in rural and urban population in southern Turkey. Exp. Parasitol..

[43] Li L.-H., Zhou X.-N., Du Z.-W., Wang X.-Z., Wang L.-B., Jiang J.-Y., Yoshikawa H., Steinmann P., Utzinger J., Wu Z. (2007). Molecular epidemiology of human Blastocystis in a village in Yunnan province, China. Parasitol. Int..

[44] Paulos S., Köster P.C., De Lucio A., Hernández-De-Mingo M., Cardona G.A., Fernández-Crespo J.C., Stensvold C.R., Carmena D. (2018). Occurrence and subtype distribution of Blastocystis sp. in humans, dogs and cats sharing household in northern Spain and assessment of zoonotic transmission risk. Zoonoses Public Health.

[45] Banhos E.F., Da Rocha J.A.M., Pimentel M.L., Batista E.T.M., Silva L.M. (2017). Prevalence and risk factors for intestinal parasite infections in schoolchildren, in the city of Santarém, Pará State, Brazil. ABCS Health Sci..

[46] Deng L., Chai Y., Zhou Z., Liu H., Zhong Z., Hu Y., Fu H., Yue C., Peng G. (2019). Epidemiology of Blastocystis sp. infection in China: A systematic review. Parasite.

[47] Lepczyńska M., Białkowska J., Dzika E., Piskorz-Ogórek K., Korycińska J. (2017). Blastocystis: How do specific diets and human gut microbiota affect its development and pathogenicity?. Eur. J. Clin. Microbiol. Infect. Dis..

[48] Pérez-Cordón G., Rosales M.J., Valdez R.A., Vargas-Vásquez F., Cordova O. (2008). Detección de parásitos intestinales en agua y alimentos de Trujillo, Perú. Rev. Peru Med. Exp. Salud Publica.

[49] Tito R.Y., Chaffron S., Caenepeel C., Lima-Mendez G., Wang J., Vieira-Silva S., Falony G., Hildebrand F., Darzi Y., Rymenans L. (2018). Population-level analysis of Blastocystis subtype prevalence and variation in the human gut microbiota. Gut.

[50] Sheehan D.J., Raucher B.G., McKitrick J.C. (1986). Association of Blastocystis hominis with signs and symptoms of human disease. J. Clin. Microbiol..

[51] Jimenez-Gonzalez D.E., Martinez-Flores W.A., Reyes-Gordillo J., Ramirez-Miranda M.E., Arroyo-Escalante S., Romero-Valdovinos M., Stark D., Souza-Saldivar V., Martinez-Hernandez F., Flisser A. (2012). Blastocystis infection is associated with irritable bowel syndrome in a Mexican patient population. Parasitol. Res..

[52] Shirvani G., Fasihi-Harandi M., Raiesi O., Bazargan N., Zahedi M.J., Sharifi I., Kalantari-Khandani B., Nooshadokht M., Shabandoust H., Mohammadi M.A. (2019). Prevalence and Molecular Subtyping of Blastocystis from Patients with Irritable Bowel Syndrome, Inflammatory Bowel Disease and Chronic Urticaria in Iran. Acta Parasitol..

[53] Dogruman-Al F., Kustimur S., Yoshikawa H., Tuncer C., Simsek Z., Tanyuksel M., Araz E., Boorom K. (2009). Blastocystis subtypes in irritable bowel syndrome and inflammatory bowel disease in Ankara, Turkey. Mem. Instit. Oswaldo Cruz.

