# Supplementary material for: Relative Frequency of Blastocystis Subtypes 1, 2, and 3 in Urban and Periurban Human Populations of Arequipa, Peru
Source: Trop Med Infect Dis. 2020 Nov 27;5(4):178. doi: 10.3390/tropicalmed5040178 (PMC7709661; doi:10.3390/tropicalmed5040178)
Supplement: Supplementary file 1 [file tropicalmed-05-00178-s001.pdf]

Tropical Medicine and Infectious Disease

Relative Frequency of *Blastocystis* Subtypes 1, 2, and 3 in Urban and Periurban Human Populations of Arequipa, Peru

Kasandra Ascuña-Durand [1,*], Renzo S. Salazar-Sánchez [1,2], Ricardo Castillo-Neyra [2,3] and Jorge Ballón-Echegaray [2,4,*]

1 Laboratorio de Microbiología Molecular, Facultad de Medicina, Universidad Nacional de San Agustín, , Arequipa, Peru; kasandra.asc@gmail.com (K.A.D.), rendaths@gmail.com (R.S.S.S.)
2 Zoonotic Disease Research Laboratory, One Health Unit, School of Public Health and Administration, Universidad Peruana Cayetano Heredia, 4314 Lima, Peru; cricardo@upenn.edu (R.C.N.), jballone@unsa.edu.pe (J.B.E.)
3 Department of Biostatistics, Epidemiology & Informatics, Perelman School of Medicine of the University of Pennsylvania, PA 19104, USA.
4 Departamento de Microbiología y Patología, Facultad de Medicina, Universidad Nacional de San Agustín, Santa Catalina 117, Arequipa, Peru; damp@unsa.edu.pe
* Correspondence: kasandra.asc@gmail.com (K.A.D.); jballone@unsa.edu.pe (J.B.E.)

Table A1. *Blastocystis* Subtypes Coinfection with Other Intestinal Protozoa.

Protozoa	Coinfection with Subtype 1	Coinfection with Subtype 3
Entamoeba coli	57.1% (4/7)	57.1% (8/14)
Chilomastix mesnilli	57.1% (4/7)	28.6% (4/14)
Giardia duodenalis	28.8% (2/7)	7.1% (1/14)
Iodamoeba bütchlii	14.3% (1/7)	21.4% (3/14)
Entamoeba histolytica	14.3% (1/7)	14.3% (2/14)
Endolimax nana	14.3% (1/7)	0% (0/14)
